# Genetic Susceptibility to Rheumatic Heart Disease: A Narrative Literature Review Highlighting Evidence From African Populations and Research Gaps in Sudan

**DOI:** 10.1002/hsr2.72283

**Published:** 2026-04-06

**Authors:** Hussam Alkhalifamohamed, Waleed M. A. Jebreel, Mohamed Elhassan, Sulafa K. M. Ali

**Affiliations:** ^1^ University of Khartoum, Khartoum, Sudan/Al Emadi Hospital Khartoum Sudan; ^2^ Institute of Endemic Diseases University of Khartoum Khartoum Sudan; ^3^ Department of Medicine RCSI University of Medicine and Health Sciences Dublin Ireland; ^4^ University of Sharjah Sharjah United Arab Emirates

**Keywords:** Africa, genetic susceptibility, GWAS, HLA, Rheumatic heart disease, Sudan

## Abstract

**Background and Aims:**

Rheumatic heart disease (RHD) remains a significant public health burden in low‐ and middle‐income countries, particularly in sub‐Saharan Africa. Although socioeconomic factors contribute to disease persistence, genetic susceptibility plays a critical role in determining progression from Group A Streptococcal infection to acute rheumatic fever (ARF) and chronic RHD. Understanding these genetic influences is essential for improving early detection and prevention strategies.

**Methods:**

A comprehensive narrative literature search was conducted using PubMed, Scopus, and Google Scholar to identify studies examining genetic susceptibility to RHD. Eligible articles included those focused on immune response genes, regional epidemiology, and genetic markers such as HLA alleles, cytokine polymorphisms, and genome‐wide association study (GWAS) findings.

**Results:**

HLA class II genes—particularly HLA‐DR and HLA‐DQ—have shown consistent associations with RHD across African, South Asian, and Latin American populations. Cytokine gene polymorphisms, including TNF‐α (−308A), IL‐6 (−174G > C), and IL‐10 (−1082G > A), have been linked to variations in inflammatory response and disease severity. GWAS have identified several novel loci, including the chromosome 11q21 region, which is unique to African cohorts, highlighting population‐specific genetic risk. Despite Sudan's high RHD burden, no dedicated genetic studies have been conducted among Sudanese patients, leaving a substantial knowledge gap regarding local susceptibility patterns.

**Conclusion:**

Current evidence underscores the importance of genetic factors in RHD pathogenesis, yet Sudan remains significantly underrepresented in genetic research. Conducting Sudan‐specific studies could identify population‐specific alleles, strengthen early screening strategies, and guide tailored prevention efforts. Such work would also contribute valuable data to the broader understanding of RHD genetics across Africa.

## Summary

- What is already known on this topic?
  ◦ Rheumatic heart disease (RHD) remains a significant health burden in low‐ and middle‐income countries, particularly in Africa. Genetic susceptibility has been suggested to influence disease development; however, limited data are available for African and Sudanese populations.
- What does this study add?
  ◦ This comprehensive literature review consolidates global genetic studies on RHD, highlights the lack of research on Sudanese populations, and underscores the importance of accounting for genetic diversity in future studies.
- How might this study affect research, practice, or policy?
  ◦ By identifying research gaps, this study can guide future genetic research in Sudan and across Africa, encourage targeted screening strategies, and contribute to global efforts to improve RHD prevention and control.

## 1 Introduction

Rheumatic heart disease (RHD) is a preventable yet life‐threatening condition caused by single or repeated episodes of acute rheumatic fever (ARF) following infection with Group A Streptococcus (GAS) [1]. It disproportionately affects populations in **low‐ and middle‐income countries (LMICs), particularly in Africa, South Asia, and the Pacific Islands** [2, 3, 4]. In Sudan, RHD remains **one of the leading causes of cardiovascular morbidity and mortality**, yet **genetic factors influencing susceptibility have not been thoroughly investigated** [5, 6, 7].

Multiple studies have demonstrated that **genetic predisposition plays a crucial role in disease susceptibility** [8, 9, 10]. While environmental and socioeconomic factors influence disease incidence [1, 3, 4, 8, 11, 12, 13], **not all individuals exposed to GAS develop RHD**, suggesting an underlying genetic component [14, 15, 16]. Familial clustering of RHD has been reported in several populations, suggesting heritable susceptibility. Studies have demonstrated increased risk among first‐degree relatives of affected individuals, supporting a genetic component to disease development. **Twin and family‐based studies** have provided strong evidence of genetic heritability, with estimates reaching **60%** [17, 18, 19].

This literature review aims to summarize key genetic findings on RHD, particularly from **African and Sudanese populations**, and to identify research gaps that must be addressed to improve disease prevention and management.

## 2 Methods

This study was conducted as a narrative review to summarize current evidence on genetic susceptibility to rheumatic heart disease (RHD), with particular emphasis on African populations and implications for Sudan. The review aimed to identify key genetic markers associated with disease susceptibility and progression, and to explore gaps in the literature relevant to high‐burden settings.

A comprehensive literature search was performed using PubMed, Scopus, and Google Scholar. Search terms included “rheumatic heart disease”, “acute rheumatic fever”, “genetic susceptibility”, “Human Leukocyte Gene (HLA)”, “cytokine polymorphism”, “genome‐wide association studies (GWAS)”, “Africa”, and “Sudan”. Studies published in English and involving human subjects were included, with no restriction on publication date. Eligible studies comprised genetic association studies, GWAS, and investigations of immune‐related polymorphisms in RHD. Animal studies, non‐English publications, and studies not addressing genetic susceptibility were excluded. Reference lists of relevant articles were also manually screened to identify additional studies.

**Figure 1:**
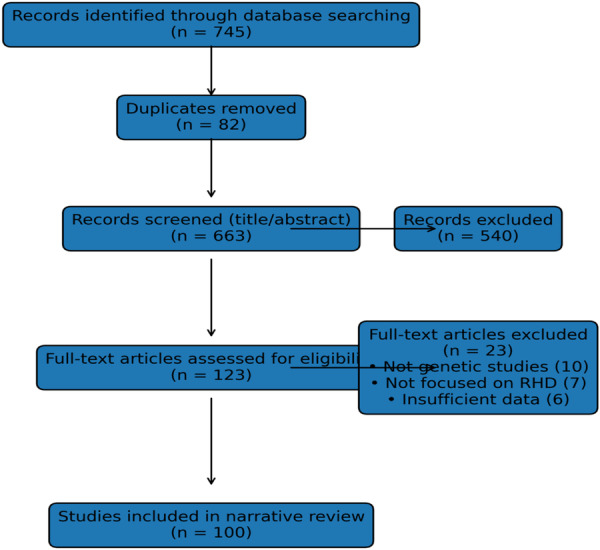
PRISMA flow diagram illustrating the literature search, screening, eligibility assessment, and inclusion of studies in this narrative review of genetic susceptibility to rheumatic heart disease.

The study selection process followed the Preferred Reporting Items for Systematic Reviews and Meta‐Analyses (PRISMA) framework. A total of 745 records were identified through database searches, of which 82 duplicates were removed. The remaining 663 articles were screened based on title and abstract, and 123 full‐text articles were assessed for eligibility. Ultimately, 100 studies were included in the final narrative synthesis (Figure 1).

Data from included studies were extracted and qualitatively synthesized. Studies were compared based on genetic markers investigated, study populations, methodological approaches, and reported associations with RHD susceptibility, severity, or outcomes. Where available, findings from multivariate analyses adjusting for potential confounders such as age, sex, socioeconomic status, and environmental exposure were also considered. The results were organized into thematic categories, including human leukocyte antigen (HLA) genes, cytokine polymorphisms, candidate genes involved in inflammatory and fibrotic pathways, and GWAS findings. This thematic synthesis enabled the integration of evidence across diverse populations while highlighting consistencies, differences, and limitations in the current literature.

## 3 Pathophysiology of Rheumatic Heart Disease

Rheumatic heart disease (RHD) results from an abnormal autoimmune response following **Group A Streptococcus (GAS)** infection, particularly pharyngitis [1, 2, 4, 11, 14, 20]. The initial phase involves **acute rheumatic fever (ARF)**, a systemic inflammatory condition affecting the heart, joints, skin, and central nervous system. If recurrent or inadequately treated, ARF can cause **permanent valvular damage**, leading to chronic RHD [1, 2, 4, 11, 12, 14, 16, 20, 21].

Several pathophysiological processes have been proposed to lead to the development of this condition. One of the most widely accepted mechanisms is **molecular mimicry**, in which the M protein of GAS shares structural similarities with human proteins, especially those found in **cardiac myosin and valve tissues**. As a result, the immune system, particularly **T and B cells, attacks host tissues and** the bacterial antigens [10, 14, 22, 23].

In genetically susceptible individuals, the immune response is amplified by the release of **proinflammatory cytokines** such as **TNF‐α, IL‐1β, IL‐10, and IFN‐γ**, leading to **valvular inflammation, fibrosis, and neovascularization** [10, 24, 25, 26, 27, 28, 29]. Chronic inflammation leads to scarring and progressive stenosis or regurgitation, particularly in the **mitral and aortic valves** [24, 27, 28, 29, 30, 31, 32].

Histological analysis of affected heart valves often shows **Aschoff bodies**—granulomatous lesions consisting of **macrophages, lymphocytes, and fibrinoid necrosis**. This infiltration leads to **chronic fibrotic remodeling** and valvular dysfunction [1, 14, 21, 22, 23, 27, 31, 33, 34, 35].

However, not all individuals exposed to GAS develop ARF or RHD, which suggests that **host genetic factors influence the strength and nature of the immune response**. Variations in genes regulating **antigen presentation (HLA), immune signaling (e.g., cytokines), and innate immunity** are believed to modulate susceptibility to RHD [8, 9, 10, 11].

## 4 Key Genetic Markers Associated with RHD

### 4.1 HLA Class II & I Genes

Genetic susceptibility to rheumatic heart disease appears to be driven primarily by variations in immune‐regulatory genes, with the most substantial evidence implicating the HLA system (Table 1). Across African, Asian, and Latin American populations, HLA class II genes—particularly HLA‐DR and HLA‐DQ—consistently emerge as significant determinants of susceptibility, reflecting their central role in antigen presentation and the initiation of autoimmune responses against cardiac tissues. While population‐specific patterns exist, such as unique risk alleles reported in South Asia, the Middle East, and East Asia, the overarching theme is that individuals with certain HLA class II configurations exhibit heightened immune reactivity to Group A Streptococcus antigens. HLA class I associations are less consistent but appear to influence disease severity in some cohorts rather than susceptibility alone [9, 10, 15, 36, 37, 38, 39, 40, 41, 42, 43, 44, 45, 46, 47, 48, 49, 50, 51, 52, 53, 54, 55, 56, 57, 58, 59, 60].

**Table 1:** Showing Various HLA Class Gene Polymorphisms.

HLA Class	Risk Allele/Variant	Protective Allele/Variant	Region/Country
Class II	DR1, DR6, DRw6	—	South Africa
Class II	DRB1*06, DRB1*11	DRB1*01, *03, *08, *10, *17	South Africa
Class II	DR11	DR1	Uganda
Class II	DRB1*0701, DQA1*0201, DRB1*13	DQA1*0201, *0103, DQB1*0603	Egypt
Class II	DQA1, DQB1, DRB	—	China, Indonesia, Pakistan, Turkey
Class II	DQA1*0101	DQA1*0102	China
Class II	DR4	—	Saudi Arabia
Class II	DR3, DR4, DR*15, DR*B4, DR*B5, DQ2	DR2	India
Class II	DRB1*03:01 (Severity)	—	India
Class II	DQA1*0104 & DQB1*05031 (MS)	—	Japan
Class II	DRB1*07, DR3, DR4 (Carditis), DR7, DR11, DRw11, Cw2	DRB1*11, 1*13, 5, 3	Turkey
Class II	DRB1*0701, DQB1*030, 1*0401‐2	—	Latvia
Class II	DR7, DRw53, DR53	—	Brazil
Class II	DR16, DR11	—	Mexico
Class II	DR1	—	Martinique
Class II	DR2 (Africans)	—	United States
Class II	DR4, DR6, DR9, & DRB1*16 (Caucasians)	—	United States
Class I	A10, B13, B16, B35	B15, B44	Turkey
Class I	B35	—	Martinique
Class I	B14, B42, Cw*4	—	Some studies

#### 4.1.1 Summary of Evidence

Overall, HLA class II alleles appear to play a significant role in susceptibility to rheumatic heart disease, supporting the importance of immune‐mediated mechanisms in disease pathogenesis.

#### 4.1.2 Consistency Across Populations

However, the specific alleles associated with increased risk vary considerably across populations, reflecting underlying genetic diversity and differences in environmental exposure to streptococcal strains.

#### 4.1.3 Limitations

These variations, along with relatively small sample sizes and heterogeneity in study design, limit the generalizability of findings and highlight the need for larger, multi‐ethnic studies.

### 4.2 Cytokine Gene Polymorphisms

Beyond the HLA system, cytokine gene polymorphisms also contribute meaningfully to genetic risk (Table 2). Variants in TNF‐α, IL‐6, IL‐10, IL‐1β, IL‐4, IL‐2, and TGF‐β1 modify the inflammatory profile of affected individuals, with risk generally linked to genotypes promoting stronger pro‐inflammatory responses or diminished anti‐inflammatory regulation. However, several cytokine associations vary across populations, suggesting that environmental exposures, ethnic genetic background, and pathogen diversity interact to shape cytokine‐related risk [9, 10, 15, 28, 61, 62, 63, 64, 65, 66, 67, 68, 69, 70, 71, 72, 73, 74].

**Table 2:** Showing Different Cytokine Gene Polymorphisms Across Populations.

Cytokine	Risk Allele/Variant	Protective Allele/Variant	Region/Country
**TNF‐α**	−308A	−308G (Mexico)	Brazil, Egypt, Mexico, Pakistan, Turkey
**TNF‐α**	−238G (Mexico)	−238A	Brazil, Mexico
**TGF‐β1**	Some variants	Some variants (Taiwan)	China, India
**IL‐1β**	−511C/T (C/T allele)	—	China, India
**IL‐1Ra**	Some alleles with MVP		Egypt, Turkey
**IL‐1**	A2	A1	Pakistan
**IL‐6**	−174G > C		Pakistan, New Zealand, Egypt (mixed results)
**IL‐10**	−1082G > A	—	Egypt
**IL‐10**	GCC Haplotype	—	Saudi Arabia
**IL‐10**	rs1800871, rs1800872	—	Russia
**IL12RB1**	rs375947	—	Russia
**IL‐4 & IL‐2**	Increased risk alleles	—	Various populations
**IL‐4**	Some variants (Latent vs. Clinical RHD studies)	—	—

#### 4.2.1 Summary of Evidence

Overall, cytokine alleles appear to play a significant role in susceptibility to rheumatic heart disease, supporting the importance of immune‐mediated mechanisms in disease pathogenesis.

#### 4.2.2 Consistency Across Populations

However, the specific alleles associated with increased risk vary considerably across populations, reflecting underlying genetic diversity and differences in environmental exposure to streptococcal strains.

#### 4.2.3 Limitations

These variations, along with relatively small sample sizes and heterogeneity in study design, limit the generalizability of findings and highlight the need for larger, multi‐ethnic studies.

### 4.3 Complement System and Other Genes

Complement pathway genes and other immune‐related loci further underscore the multi‐pathway nature of genetic susceptibility (Table 3). Variants in Mannose Binding Lectin (MBL2) and Mannose‐Associated Serine Protease (MASP‐2) highlight the lectin pathway's involvement, while Angiotensin‐converting Enzyme (ACE) polymorphisms are consistently associated with valvular calcification and disease progression. Toll‐like receptor (TLR) polymorphisms implicate disrupted innate immune recognition, and recent findings on Filicon (FCN1) promoter variants reveal a dual biological role—providing protection in the acute phase of rheumatic fever yet contributing to progressive valvular damage in chronic disease [9, 10, 15, 63, 64, 74, 75, 76, 77, 78, 79, 80, 81, 82, 83, 84, 85, 86].

**Table 3:** Showing Complement and Other Genes' Polymorphism Across Populations.

Gene/Factor	Risk Allele/Variant	Protective Allele/Variant	Region/Country
**MBL2**	A/A Genotype		Brazil
**MMP1**	rs1799750		China
**MASP‐2**	Some haplotypes	Some haplotypes	Brazil
**TLR**	R1, R2, R4, R5, R6	—	China, India, Russia, Turkey
**STAT**	3, 5B	—	India
**BNP**	CpG4, CpG5	—	China
**FMF**	FMF Gene	—	Turkey
**Ficolin‐1 (FCN1)**	Minor Promoter variants (valve damage)	—	—
**Ficolin‐2 (FCN2)**	AGGT Haplotype	GGAC Haplotype	India
**Ficolin‐3 (FCN3)**	Variant A Allele (rs4494157)	—	Egypt
**AGTR1**	AC Allele	AA and CC Alleles	—
**ACE**	DD & II Alleles	ID & DD Alleles	Taiwan & others
**CTLA‐4**	Depleted in MVL & CVL	—	India
**CRP**	rs1130864, rs1205, rs3093077		Russia

#### 4.3.1 Summary of Evidence

Genetic polymorphisms in complements and other inflammatory pathway genes consistently suggest a role in modulating susceptibility and disease severity in rheumatic heart disease.

#### 4.3.2 Consistency Across Populations

Nevertheless, associations are not uniform across studies, with some polymorphisms demonstrating population‐specific effects.

#### 4.3.3 Limitations

The variability in findings may be attributed to differences in study populations, sample sizes, and gene–environment interactions, underscoring the need for standardized and larger‐scale investigations.

### 4.4 GWAS and Novel Loci

Genome‐wide association studies (GWAS) have expanded understanding beyond candidate‐gene frameworks, revealing population‐specific loci that underline the heterogeneity of RHD genetics (Table 4). A large pan‐African GWAS that included participants from multiple African countries, including limited representation from Sudan, identified a novel susceptibility locus at chromosome 11q21. However, no study has specifically analyzed genetic susceptibility to RHD in a Sudanese cohort or reported Sudan‐specific genetic associations. Other GWAS in Australian Aboriginal, Pacific Islander, and South Asian populations identified significant associations in the HLA‐DQA1 region, Immunoglobulin Heavy Variable (IGHV) genes, and Pre‐B‐cell leukemia homeobox 2 (PBX2), respectively. Collectively, these findings might demonstrate that while certain immunogenetic mechanisms are conserved across populations, others are uniquely shaped by ancestry, reinforcing the need for region‐specific genomic studies—particularly in Sudan, where no genetic research on RHD has been conducted to date [9, 87, 88, 89, 90].

**Table 4:** Showing GWAS Conducted in Different Populations.

Region/Country	GWAS Locus
Africa	Chromosome 11q21
Aboriginal Australians	HLA‐DQA1 (rs9272622)
Pacific Islanders	IGHV4‐61*02
South Asia	PBX2 (HLA Class III region)

#### 4.4.1 Summary of Evidence

Genome‐wide association studies have identified novel susceptibility loci, providing important insights into the genetic architecture of rheumatic heart disease beyond candidate gene approaches.

#### 4.4.2 Consistency Across Populations

Notably, some loci appear to be population‐specific, particularly in African cohorts, highlighting the importance of studying diverse populations.

#### 4.4.3 Limitations

However, the limited number of GWAS conducted in African populations, including Sudan, restricts the ability to draw definitive conclusions and emphasizes the need for further large‐scale genomic studies.

### 4.5 Genetic Markers and Disease Severity

Beyond disease susceptibility, several studies have investigated whether genetic polymorphisms influence the severity and clinical outcomes of rheumatic heart disease. Variants in inflammatory cytokine genes such as **TNF‐α, IL‐6, and IL‐10** have been associated with stronger inflammatory responses and may contribute to more aggressive valvular damage. Similarly, polymorphisms in the **angiotensin‐converting enzyme (ACE)** gene have been linked to increased valvular fibrosis and calcification, suggesting a potential role in structural disease progression. However, the evidence remains heterogeneous, and most studies are limited by small sample sizes and population‐specific findings. Large multicenter studies are needed to clarify the relationship between genetic susceptibility and disease severity in RHD.

## 5 Sudanese and African Data

Sudan carries one of the highest documented burdens of rheumatic heart disease (RHD) in Africa, with a striking heterogeneity across regions. Echocardiographic surveys consistently reveal clustered pockets of extremely high prevalence, far exceeding national and regional averages. School‐based screening studies have demonstrated prevalence rates ranging from **0.3 per 1000 in Khartoum** to **19 per 1000 in Niyala (South Darfur)** [91] and an alarming **61 per 1000 in North Kordofan** [92], underscoring profound geographic and socioeconomic disparities. These findings align with regional analyses: a 2023 meta‐analysis of East Africa estimated Sudan's pooled RHD prevalence at **3% (95% CI: 1–10%)** [93], markedly lower than Ethiopia's but higher than Tanzania's, yet Sudan's internal variation remains among the most extreme in the region.

Hospital‐based data further illustrate the severity of the disease at presentation. In North Darfur, 83% of patients seen at Al‐Fashir Hospital had severe valve involvement, over half had more than two valves affected, and inpatient mortality reached **12%** [94], emphasizing the consequences of late detection and limited access to secondary prophylaxis. Additional studies in conflict‐affected regions, such as West and North Darfur, reveal prevalences of 16–17 per 1000, more than five times higher than in relatively stable regions like Kassala (3.1 per 1000) [95], suggesting a strong interplay between socioeconomic vulnerability, healthcare fragmentation, and disease clustering.

Sudan has implemented national RHD control initiatives such as the Sudanese RHD control program (SUR I CAAN), which has been among the most active in the region and provides a platform for clinical screening and registry development. These initiatives could facilitate future genetic epidemiology studies through the establishment of patient cohorts and biological sample collection. Since 2012, the *SUR I CAAN* and “Research for Life” initiatives have integrated handheld echocardiographic screening, school‐based programs, health worker training, and targeted awareness campaigns. More than **12,000 children** have been screened across multiple states, with hundreds of health workers trained and localized guideline implementation strengthened [90]. Despite these advances, challenges persist, including inconsistent benzathine penicillin supply, health‐system instability, and uneven training across rural areas. The 2022 paradigm‐shift recommendations from Sudanese cardiology leaders advocate lowering diagnostic thresholds, adopting screen‐to‐treat approaches, and expanding primary prevention strategies—critical steps in light of recent evidence showing that handheld echocardiography can identify substantial burdens of *silent acute rheumatic fever* and latent RHD [96].

At the severe end of the spectrum, the Salam Centre for Cardiac Surgery in Khartoum has performed over **8000 free cardiac surgeries**—the largest RHD surgical series in sub‐Saharan Africa—demonstrating both the scale of Sudan's disease burden and the feasibility of high‐quality care in resource‐limited settings. Five‐year survival after surgery exceeds **85%**, but the young age of patients and the rate of multiple valve involvement underscore failures of early detection and prevention [97].

Despite Sudan's exceptional burden, no published genetic studies have examined RHD susceptibility, HLA associations, cytokine polymorphisms, or genomic architecture in Sudanese populations. This is particularly striking given Sudan's **extraordinary genetic diversity**, shaped by centuries of Afro‐Arab admixture and population movement across the Sahel corridor [98, 99, 100]. Data from Uganda, Egypt, and South Africa reveal clear population‐specific genetic patterns—yet the applicability of these findings to Sudan is unknown [40, 41, 59, 61, 62, 75]. Given the pronounced regional clustering of disease, variable prevalence across ethnic groups, and the presence of high‐burden “hotspots,” Sudan is a uniquely important setting for genomic investigation. Conducting GWAS or targeted genetic studies in Sudanese cohorts offers the potential not only to identify population‐specific risk alleles but also to illuminate gene‐environment interactions contributing to the extreme geographic disparities observed.

Sudan represents a uniquely important setting for genetic research in rheumatic heart disease (RHD) due to the convergence of several key factors. The country bears a disproportionately high disease burden, often with early onset and severe clinical presentations, increasing the likelihood of detecting meaningful genetic associations. Sudanese populations also exhibit substantial genetic diversity and complex ancestral backgrounds, which may facilitate the identification of novel susceptibility loci not observed in more homogeneous populations. In addition, persistent environmental exposures—particularly recurrent group A streptococcal infections and adverse socioeconomic conditions—provide a valuable context for investigating gene–environment interactions. Importantly, existing national RHD control and screening programs offer a practical infrastructure for cohort establishment, systematic data collection, and future integration of genetic studies. Despite these advantages, Sudan remains largely absent from genomic research, representing a critical gap in African cardiovascular genetics. Addressing this gap would not only advance understanding of RHD pathogenesis but also support the development of population‐specific risk stratification and precision prevention strategies, with potential relevance to other high‐burden regions.

## 6 Gaps in Research and Future Directions

Although Sudan contributes significantly to the regional burden of rheumatic heart disease, its genetic landscape remains largely unexplored. Although Sudanese individuals have been included in broader African GWAS, no studies have specifically investigated genetic susceptibility to RHD within Sudanese populations. However, the absence of Sudan‐specific subgroup analysis means that no study has yet clarified how genetic variation uniquely influences RHD susceptibility or severity within Sudan's diverse ethnic groups, leaving significant gaps in understanding genotype–phenotype relationships. A focused Sudanese genetic study could identify population‐specific alleles, elucidate correlations between genetic markers and disease severity, and improve understanding of gene–environment interactions in a setting characterized by marked socioeconomic and geographic disparities. Such work would fill a critical regional research gap, contribute to global efforts to define the genetic architecture of RHD, and ultimately support the development of targeted prevention strategies, more accurate early screening, and personalized treatment approaches for Sudanese patients.

## 7 Conclusion

Rheumatic heart disease remains a significant cause of preventable cardiovascular morbidity across Africa, with Sudan represented among the highest‐burden countries. Global research has established a substantial genetic contribution to RHD, involving HLA alleles, cytokine polymorphisms, complement pathway genes, and multiple novel loci identified through GWAS. However, the absence of Sudan‐specific genetic studies represents a significant gap, particularly given the country's unique ancestral composition and high disease prevalence. Filling this gap through robust genomic research will be essential to understanding population‐specific susceptibility, improving early detection, refining risk stratification, and guiding precision prevention strategies. Ultimately, expanding genetic research in Sudan and across Africa will contribute not only to regional disease control but also to the broader global effort to unravel the complex immunogenetic pathways that underlie rheumatic heart disease.

Bridging these research gaps holds significant potential to inform precision prevention, enhance early screening programs, and support more efficient allocation of limited healthcare resources for populations at highest risk in Sudan and across Africa. Ultimately, advancing genetic research in Sudan has the potential to transform the current approach to rheumatic heart disease from reactive management to proactive, risk‐based prevention.

## Author Contributions

**Hussam Alkhalifamohamed:** searched the databases, wrote the initial and final draft. **Waleed M. A. Jebreel:** reviewed the draft critically, agreed on the papers to be included, and did the citations. **Mohamed Elhassan:** reviewed the draft critically, agreed on the papers to be included, checked the language, and checked for plagiarism. **Sulafa K. M. Ali:** supervised the whole process (our supervisor).

## Funding Statement

The authors have nothing to report.

## Conflicts of Interest

The authors declare no conflicts of interest.

## Transparency Statement

The lead author Hussam Alkhalifamohamed affirms that this manuscript is an honest, accurate, and transparent account of the study being reported; that no important aspects of the study have been omitted; and that any discrepancies from the study as planned (and, if relevant, registered) have been explained.

## Data Availability

The data supporting the findings of this study are available within the article and its supplementary materials.
